# The Correlations Between Plasma Fibrinogen With Amyloid-Beta and Tau Levels in Patients With Alzheimer’s Disease

**DOI:** 10.3389/fnins.2020.625844

**Published:** 2021-01-21

**Authors:** Dong-Yu Fan, Hao-Lun Sun, Pu-Yang Sun, Jie-Ming Jian, Wei-Wei Li, Ying-Ying Shen, Fan Zeng, Yan-Jiang Wang, Xian-Le Bu

**Affiliations:** ^1^Department of Neurology and Centre for Clinical Neuroscience, Daping Hospital, Third Military Medical University, Chongqing, China; ^2^Shigatse Branch, Xinqiao Hospital, Third Military Medical University, Shigatse, China

**Keywords:** Alzheimer’s disease, fibrinogen, β-amyloid, tau, pathogenesis, biomarkers

## Abstract

Recent studies show that fibrinogen plays a role in the pathogenesis of Alzheimer’s disease (AD), which may be crucial to neurovascular damage and cognitive impairment. However, there are few clinical studies on the relationship between fibrinogen and AD. 59 ^11^C-PiB-PET diagnosed AD patients and 76 age- and gender-matched cognitively normal controls were included to analyze the correlation between plasma β-amyloid (Aβ) and tau levels with fibrinogen levels. 35 AD patients and 76 controls with cerebrospinal fluid (CSF) samples were included to further analyze the correlation between CSF Aβ and tau levels with fibrinogen levels. In AD patients, plasma fibrinogen levels were positively correlated with plasma Aβ40 and Aβ42 levels, and negatively correlated with CSF Aβ42 levels. Besides, fibrinogen levels were positively correlated with CSF total tau (t-tau), and phosphorylated tau-181 (p-tau) levels and positively correlated with the indicators of Aβ deposition in the brain, such as t-tau/Aβ42, p-tau/Aβ42 levels. In normal people, fibrinogen levels lack correlation with Aβ and tau levels in plasma and CSF. This study suggests that plasma fibrinogen levels are positively correlated with Aβ levels in the plasma and brain in AD patients. Fibrinogen may be involved in the pathogenesis of AD.

## Introduction

Alzheimer’s disease (AD) is the most common neurodegenerative disease that causes cognitive and memory impairment (Castellani et al., 2010; Jia et al., 2014). The main pathological hallmarks of AD include extracellular senile plaques containing β-amyloid (Aβ) and intracellular neurofibrillary tangles formed by phosphorylated tau (Huang and Mucke, 2012; Long and Holtzman, 2019). Recent studies have shown that fibrinogen also plays an important role in the pathogenesis of AD (Cortes-Canteli and Strickland, 2009). Fibrinogen can bind to Aβ, which intensifies inflammation in the AD brain and accelerates the decline of cognitive function in AD patients (Ahn et al., 2014; Merlini et al., 2019). The Aβ-fibrinogen interaction may be crucial to the progression of neurovascular damage and cognitive impairment in AD (Xu et al., 2008; Cortes-Canteli et al., 2010). However, there are few clinical studies on the relationship between fibrinogen and Aβ. This study aims to explore the relationship between fibrinogen and Aβ levels in AD patients and normal people.

## Materials and Methods

### Study Population

Alzheimer’s disease patients were recruited from Chongqing Daping Hospital from December 2018 to May 2020. Age- and gender-matched controls with normal cognition were randomly recruited from the hospital at the same time. Subjects were excluded for the following reasons: (1) a family history of dementia; (2) a concomitant neurologic disorder that could potentially affect the cognitive function or other types of dementia; (3) severe cardiac, pulmonary, hepatic, or renal diseases or any type of tumor; (4) enduring mental illness (e.g., schizophrenia); (5) Diseases that may affect fibrinogen levels (e.g., bleeding disorders, hereditary abnormal fibrinogenemia, etc.); (6) Recently used treatments that affect fibrinogen levels (e.g., blood transfusion); (7) an allergy to the ^11^C-Pittsburgh compound.

### AD Diagnosis and Sampling

The diagnosis of AD was made according to the criteria of the National Institute of Neurological and Communicative Diseases and Stroke/AD and Related Disorders Association following the protocols we used before (Li et al., 2011). Besides, the patients who collected blood all received Aβ positron emission tomography (PET) examination of Pittsburgh compound B (PiB), and the diagnostic criteria were PiB-PET positive. The demographic data and medical history (such as hypertension, coronary heart disease, and diabetes mellitus) were collected and the cognitive and functional status was assessed based on a neuropsychological battery. Fasting blood was collected between 07:00 and 09:00 to avoid the potential circadian rhythm influence. The blood samples were centrifuged within an hour of collection and EDTA plasma was aliquoted in 0.5 mL polypropylene tubes and stored at −80°C until used. The cerebrospinal fluid (CSF) samples were centrifuged at 2,000*g* at 4°C for 10 min, and the aliquots were then immediately frozen and stored at −80°C until use. The informed consent was obtained before the acquisition of the blood and CSF samples.

### Measurements of Fibrinogen, Aβ, and Tau Levels

Fibrinogen levels were measured using standard laboratory methods in the Clinical Laboratory, Daping Hospital, Chongqing, China. Fibrinogen−C is the test to measure fibrinogen by the Clasus method and is carried out with the commercial kit HemosIL Fibrinogen assay (Instrumentation Laboratory Company, United States) on ACL-TOP (Instrumentation Laboratory Company, United States). The kit uses an excess of thrombin to convert fibrinogen to fibrin in diluted plasma. Plasma levels of Aβ42, Aβ40 were measured using the commercially available single-molecule array (SIMOA) Human Neurology 3-Plex A assay kit (Quanterix, United States) on-board of the automated SIMOA HD-1 analyzer (Quanterix, United States). CSF levels of Aβ40, Aβ42, total tau (t-tau), and phosphorylated tau-181 (p-tau) were measured using the human Aβ and tau enzyme-linked immunosorbent assay (ELISA) kits (Innotest, United States). All of the measurements were performed according to the manufacturer’s instructions (Wilke et al., 2018).

### Statistical Analysis

The differences in demographic characteristics and fibrinogen levels between the groups were assessed with two-tailed independent *t*-tests, Mann–Whitney *U* test, or Chi-square test. Spearman correlation analyses were used to examine the correlations between fibrinogen levels and Aβ levels. The data are expressed as the mean ± standard deviation (SD). All hypothesis testing was two-sided, and *p* < 0.05 was defined as statistically significant. The computations were performed with SPSS version 20.0 (SPSS Inc., United States).

## Results

### Characteristics of the Study Population

The characteristics of the subjects are shown in Tables 1, 2. The study consisted of 59 AD patients diagnosed by ^11^C-PiB PET and 76 age- and gender-matched cognitively normal controls. There were no significant differences in age, sex, education level, or the comorbidity of hypertension, diabetes mellitus, cardiovascular disease, and hyperlipidemia between AD patients and cognitively normal controls. AD patients consisted of a higher proportion of APOE ε4 carriers (*p* = 0.004) and showed lower MMSE scores (*p* < 0.001). The AD patients had lower levels of both plasma Aβ40 (219.2 ± 107.1 pg/mL vs. 284.4 ± 71.67 pg/mL, *p* < 0.001) and Aβ42 (9.915 ± 5.126 pg/mL vs. 15.42 ± 4.598 pg/mL, *p* < 0.001) than the control group. The AD patients with CSF had lower levels of CSF Aβ40 (9150 ± 3926 pg/mL vs. 12190 ± 4482 pg/mL, *p* = 0.001) and Aβ42 (629.5 ± 286.5 pg/mL vs. 1508 ± 673.2 pg/mL, *p* < 0.001), and higher levels of CSF t-tau (402.3 ± 183.6 pg/mL vs. 184.0 ± 61.38 pg/mL, *p* < 0.001), CSF p-tau (66.09 ± 28.38 pg/mL vs. 42.84 ± 18.18 pg/mL, *p* < 0.001), CSF t-tau/Aβ42 (0.809 ± 0.511 pg/mL vs. 0.146 ± 0.080 pg/mL, *p* < 0.001), and CSF t-tau/Aβ42 (0.1317 ± 0.0844 pg/mL vs. 0.0334 ± 0.0206 pg/mL, *p* < 0.001) than the control group.

**TABLE 1:** Characteristics of the participants with plasma samples.

Characteristics	Controls (*n* = 76)	PiB-PET (+)-AD (*n* = 59)	*p-*value
Age, mean (SD), y	68.42 (8.52)	66.31 (9.53)	0.180
Female, *n* (%)	46 (60.5)	33 (57.6)	0.602
Education level, mean (SD), *y*	9.24 (4.36)	9.61 (4.44)	0.629
MMSE score, mean (SD)	26.28 (3.05)	12.37 (5.06)	<0.001
APOE ε4 carriers, no (%)	8 (10.53)	18 (30.51)	0.004
Diabetes, (%)	11 (14.47)	9 (15.25)	>0.999
Hypertension, (%)	19 (25.00)	15 (25.42)	>0.999
Dyslipidaemia, (%)	21 (27.63)	16 (27.12)	>0.999
Coronary artery disease, (%)	13 (17.11)	11 (18.64)	0.825
Stroke history, (%)	6 (7.89)	3 (5.08)	0.731
Plasma Aβ40, mean (SD), pg/mL	284.4 (71.67)	219.2 (107.1)	<0.001
Plasma Aβ42, mean (SD), pg/mL	15.42 (4.598)	9.915 (5.126)	<0.001
Plasma t-tau, mean (SD), pg/mL	4.544 (2.536)	5.923 (3.196)	0.006
Plasma t-tau/Aβ42, mean (SD), pg/mL	0.3271 (0.2658)	0.8143 (0.8529)	<0.001
Plasma Aβ42/Aβ40, mean (SD), pg/mL	0.05516 (0.01391)	0.04859 (0.01793)	0.018

*MMSE, mini-mental state examination; APOE ε4, apolipoprotein E ε4 allele; *p*-value, two-tailed independent *t*-tests, Mann–Whitney *U* test or Chi-square test as appropriate.*

**TABLE 2:** Characteristics of the participants with CSF samples.

Characteristics	Controls (*n* = 76)	AD (*n* = 35)	*p-*value
Age, mean (SD), *y*	68.42 (8.52)	66.34 (1.47)	0.240
Female, *n* (%)	46 (60.5)	18 (51.43)	0.412
Education level, mean (SD), *y*	9.24 (4.36)	9.63 (3.80)	0.652
MMSE score, mean (SD)	26.28 (3.05)	12.03 (4.08)	<0.001
APOE ε4 carriers, No (%)	8 (10.53)	14 (40.00)	0.001
Diabetes, (%)	11 (14.47)	6 (17.14)	0.779
Hypertension, (%)	19 (25.00)	8 (22.86)	0.819
Dyslipidaemia, (%)	21 (27.63)	11 (31.43)	0.822
Coronary artery disease, (%)	13 (17.11)	7 (20.00)	0.792
Stroke history, (%)	6 (7.89)	2 (5.71)	0.726
CSF Aβ40, mean (SD), pg/mL	12190 (4482)	9150 (3926)	0.001
CSF Aβ42, mean (SD), pg/mL	1508 (673.2)	629.5 (286.5)	<0.001
CSF t-tau, mean (SD), pg/mL	184.0 (61.38)	402.3 (183.6)	<0.001
CSF p-tau, mean (SD), pg/mL	42.84 (18.18)	66.09 (28.38)	<0.001
CSF t-tau/Aβ42, mean (SD), pg/mL	0.146 (0.080)	0.8867 (0.672)	<0.001
CSF p-tau/Aβ42, mean (SD), pg/mL	0.0334 (0.0206)	0.1317 (0.0844)	<0.001

*MMSE, mini-mental state examination; APOE ε4, apolipoprotein E ε4 allele; *p*-value, two-tailed independent *t*-tests, Mann–Whitney *U* test or Chi-square test as appropriate.*

### Correlation Between Fibrinogen Levels With Plasma Aβ Levels

**FIGURE 1:**
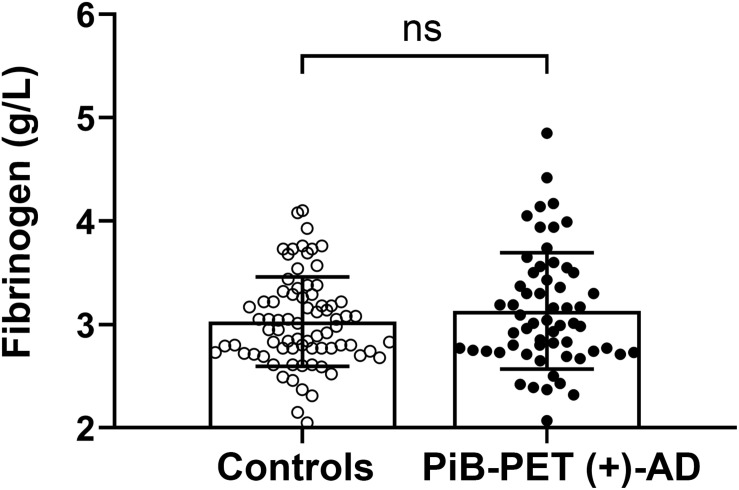
Comparison of the plasma fibrinogen levels between the controls and PiB-PET (+)-AD patients. ns denotes no statistical significance.

There was no significant difference in plasma fibrinogen levels between AD patients and the control group [PiB-PET (+)-AD vs controls: 3.13 ± 0.563 g/L vs. 3.03 ± 0.433 g/L, *p* = 0.256] (Figure 1). There was also no significant difference in fibrinogen levels between APOE ε4 carriers and APOE ε4 non-carriers (Supplementary Figure 1A). Besides, there was no significant correlation between fibrinogen levels and MMSE scores (Supplementary Figure 1B).

**FIGURE 2:**
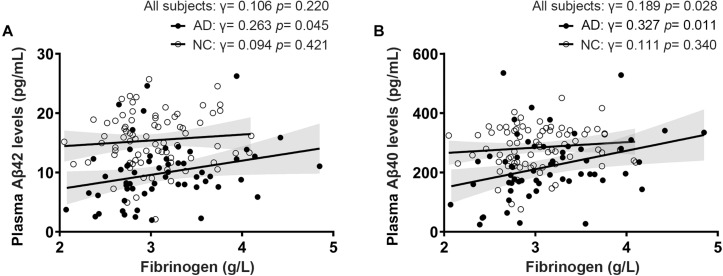
Correlations between fibrinogen levels with plasma Aβ42 levels **(A)** and Aβ40 levels **(B)** in AD patients diagnosed by positive PiB-PET and normal controls (NC).

Fibrinogen levels in AD patients diagnosed by positive PiB-PET had a significantly positive correlation with plasma Aβ42 levels (γ = 0.263, *p* = 0.045) and Aβ40 levels (γ = 0.327, *p* = 0.011). There was no correlation between fibrinogen levels and plasma Aβ42 levels (γ = 0.094, *p* = 0.421) and Aβ40 levels (γ = 0.111, *p* = 0.340) in controls. In all subjects, fibrinogen levels had a significantly positive correlation with plasma Aβ40 levels (γ = 0.189, *p* = 0.028) but not with Aβ42 levels (γ = 0.106, *p* = 0.220) (Figure 2). There was no correlation between fibrinogen levels in both AD and controls with plasma t-tau levels, Aβ42/Aβ40 levels, and t-tau/Aβ42 levels (Supplementary Figure 2).

### Correlation Between Fibrinogen Levels With CSF Aβ Levels

**FIGURE 3:**
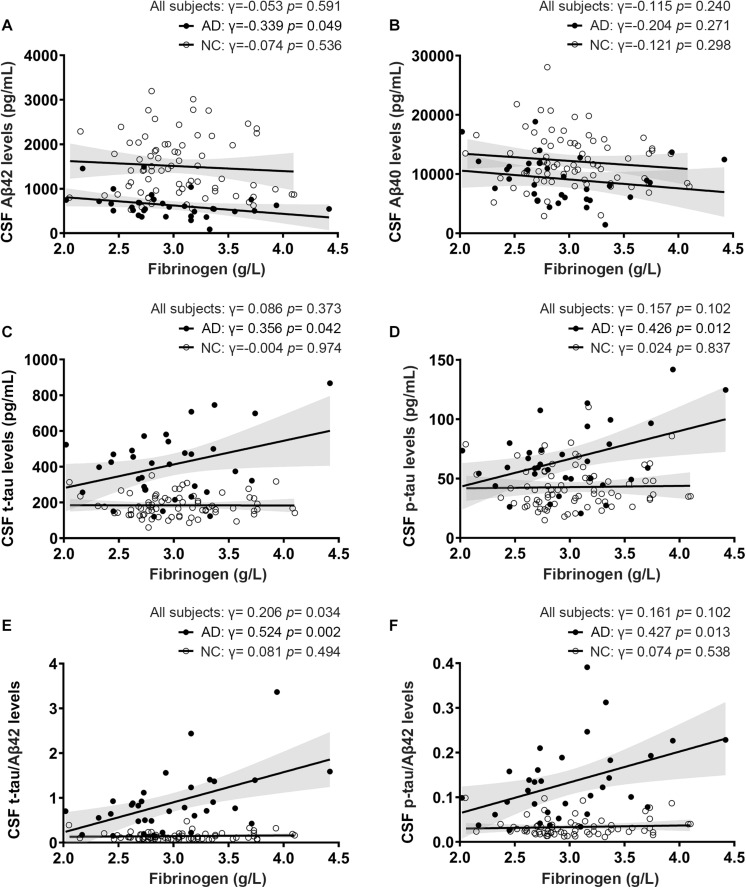
Correlations between fibrinogen levels with CSF Aβ levels **(A,B)**, tau levels **(C,D)**, and tau/Aβ42 levels **(E,F)** in AD patients and normal controls (NC).

Of all the clinical AD patients, 35 people had CSF collected to further analyze the correlation between fibrinogen levels with CSF Aβ and tau levels. As shown in Table 2, there were no significant differences in the comorbidity of hypertension, diabetes mellitus, cardiovascular disease, and hyperlipidemia between the two groups. Also, no significant difference was found in the fibrinogen between these two groups (AD vs controls: 2.97 ± 0.510 g/L vs. 3.03 ± 0.433 g/L, *p* = 0.541). Fibrinogen levels in AD patients had significantly positive correlation with CSF Aβ42 levels (γ = −0.339, *p* = 0.049), but no correlation with CSF Aβ40 levels (γ = −0.204, *p* = 0.271). There was no correlation between fibrinogen levels in controls with CSF Aβ42 levels (γ = −0.074, *p* = 0.536) and Aβ40 levels (γ = −0.121, *p* = 0.298). In all subjects, there was no correlation between fibrinogen levels with CSF Aβ42 levels (γ = −0.053, *p* = 0.591) and Aβ40 levels (γ = −0.115, *p* = 0.240) (Figures 3A,B).

### Correlation Between Fibrinogen Levels With CSF Tau Levels

To further reveal the relationship between fibrinogen and AD pathological changes, we then detected the t-tau and phosphorylated tau in CSF and analyzed their correlation. Fibrinogen levels in AD patients had significantly positive correlation with CSF t-tau levels (γ = 0.356, *p* = 0.042) and p-tau levels (γ = 0.426, *p* = 0.012). There was no correlation between fibrinogen levels in controls with CSF t-tau levels (γ = −0.004, *p* = 0.974) and p-tau levels (γ = 0.024, *p* = 0.837). In all subjects, there was no correlation between fibrinogen levels with CSF t-tau levels (γ = 0.086, *p* = 0.373) and p-tau levels (γ = 0.157, *p* = 0.102) (Figures 3C,D).

### Correlation Between Fibrinogen Levels With CSF Tau/Aβ42 Levels

Compared with a single marker, recent studies have found that the ratio of tau and Aβ42, including t-tau/Aβ42 and p-tau/Aβ42, has a higher correlation with the PiB-PET cortical standard uptake ratio (SUVR), which can better reflect the pathology of Aβ deposition in the brain (Hansson et al., 2018; Schindler et al., 2018). Based on this, we calculated the correlation between these two ratios and fibrinogen to explore the relationship between fibrinogen and the pathological process in the brain. We found that fibrinogen levels in AD patients had significantly positive correlation with CSF t-tau/Aβ42 levels (γ = 0.524, *p* = 0.002) and p-tau/Aβ42 levels (γ = 0.427, *p* = 0.013). There was no correlation between fibrinogen levels in controls with CSF t-tau/Aβ42 levels (γ = 0.081, *p* = 0.494) and p-tau/Aβ42 levels (γ = 0.074, *p* = 0.538). In all subjects, fibrinogen levels had a significantly positive correlation with CSF t-tau/Aβ42 levels (γ = 0.206, *p* = 0.034) but not with p-tau/Aβ42 levels (γ = 0.161, *p* = 0.102) (Figures 3E,F).

## Discussion

This study explored the correlation between fibrinogen levels and Aβ, tau levels in humans for the first time. In AD patients, fibrinogen levels were positively correlated with plasma Aβ40 and Aβ42 levels, and negatively correlated with CSF Aβ42 levels. Besides, fibrinogen levels were positively correlated with CSF t-tau and p-tau levels and were positively correlated with the indicators of Aβ deposition in the brain, such as t-tau/Aβ42, p-tau/Aβ42 levels. In normal people, fibrinogen levels lack correlation with Aβ and tau levels in plasma and CSF.

Previous studies have shown that the destruction of the blood-brain barrier can cause fibrinogen to enter the brain and accelerate neuronal damage in the pathological process of neurological diseases such as AD (Adams et al., 2004). Therefore, compared with normal people, the pathological development may be aggravated due to a large amount of fibrinogen in the brain of AD patients (Lipinski and Sajdel-Sulkowska, 2006; Cortes-Canteli et al., 2014), and the cognitive function of AD patients decreases as their plasma fibrinogen levels increase (Oijen et al., 2006; Xu et al., 2008). In this study, we found that plasma and CSF Aβ levels in AD patients were significantly correlated with their plasma fibrinogen levels, which further provided clinical evidence that fibrinogen may involve in the development of AD pathological damage.

Platelets are the main place where Aβ is produced in the periphery, so the activation of platelets will increase the production of peripheral Aβ (Chen et al., 1995; Shen et al., 2008). Fibrinogen can induce platelet aggregation and activation, leading to more blood Aβ formation (Bennett, 2001; Chen et al., 2003). The fibrinogen in the brain of AD patients will combine with Aβ deposition to form oligomers with abnormal structures, resulting in a decrease of free fibrinogen levels in the plasma (Ahn et al., 2010). This is also the possible reason why the plasma fibrinogen in AD patients is not significantly increased. These oligomers are difficult to degrade, they can block blood vessels, cause thrombosis and abnormal fibrinolysis, reduce cerebral blood flow perfusion, accelerate neurovascular injury and neuroinflammation, and aggravate the formation of amyloid angiopathy (CAA) (Paul et al., 2007; Cortes-Canteli et al., 2010). The increased binding affinity of Aβ to fibrinogen will aggravate the above process and lead to the occurrence of hereditary cerebral amyloid angiopathy (HCAA) (Cajamarca et al., 2020). In addition to forming complexes with Aβ, fibrinogen in cerebral blood vessels will also form clots with the help of APOE ϵ4 gene and homocysteine, leading to more Aβ deposition in the cerebral blood vessel wall and CAA formation (Hultman et al., 2013; Chung et al., 2016). Besides, too much fibrinogen in the brain can also lead to insufficient cerebral perfusion, aggravating cerebral hypoxia, and the formation of Aβ plaques in AD patients (Miners et al., 2018). Animal studies have also observed that removing this part of fibrinogen can alleviate AD-related pathologies in the brain of mice and improve cognitive impairment (Cortes-Canteli et al., 2010). Therefore, there is a positive correlation between plasma Aβ and fibrinogen levels, and a negative correlation between CSF Aβ and fibrinogen levels in AD patients, and the more stable Aβ42 has a better correlation, while the integrity of the blood-brain barrier in normal people is not destroyed, which may be the reason for the lack of correlation between them.

According to the Aβ cascade hypothesis, the increase of Aβ can further induce the hyperphosphorylation of the microtubule-associated protein tau and accumulation in the cells, forming AD-related pathological changes such as neurofibrillary tangles, and leading to increased levels of t-tau and p-tau in the CSF of AD patients (Huang and Mucke, 2012). This may explain the positive correlation between tau and fibrinogen levels in AD patients. But so far there is no direct evidence that fibrinogen can exacerbate tau phosphorylation.

In addition to the Aβ pathway, previous studies have also found that fibrinogen can directly affect neuroinflammation by inducing the activation of microglia through CD11b/CD18 integrin receptors and other means (Ryu et al., 2009). Blocking this approach can reduce neuroinflammation, synaptic dysfunction, and cognitive decline in AD mice (Merlini et al., 2019). Fibrinogen may play an important role in the pathogenesis of AD.

This study provides clinical evidence for the relationship between fibrinogen and AD, suggesting that fibrinogen may play a role in the pathogenesis of AD. It is worth noting that this is a cross-sectional observational study, we cannot determine the effect of fibrinogen on the progression of AD. To further clarify the impact, cohort studies need to be continued in the future. In addition, we need to increase the number of CSF samples from AD patients, adopt more accurate detection methods for CSF biomarkers such as SIMOA, and further analyze the correlation between fibrinogen and amyloid-PET SUVR to better verify the effect of fibrinogen on Aβ deposition in the brain. At the same time, whether drugs to reduce fibrinogen will improve the cognitive function decline of AD patients remains to be further studied.

## Conclusion

Our research shows that plasma fibrinogen levels are positively correlated with Aβ levels in the plasma and brain in AD patients, which further shows that fibrinogen can promote Aβ deposition in the brain and accelerate tau phosphorylation. Fibrinogen may be involved in the pathogenesis of AD.

## Data Availability Statement

The raw data supporting the conclusions of this article will be made available by the authors, without undue reservation.

## Ethics Statement

The studies involving human participants were reviewed and approved by the Institutional Review Board of Daping Hospital. The patients/participants provided their written informed consent to participate in this study.

## Author Contributions

X-LB, Y-JW, and D-YF designed this study. D-YF, W-WL, J-MJ, and Y-YS performed biomarker testing and clinical the data collection. H-LS, D-YF, P-YS, and FZ analyzed the data. D-YF and H-LS wrote the article. All authors contributed to the article and approved the submitted version.

## Conflict of Interest

The authors declare that the research was conducted in the absence of any commercial or financial relationships that could be construed as a potential conflict of interest.
