# Supplementary material for: The Correlations Between Plasma Fibrinogen With Amyloid-Beta and Tau Levels in Patients With Alzheimer’s Disease
Source: Front Neurosci. 2021 Jan 21;14:625844. doi: 10.3389/fnins.2020.625844 (PMC7859103; doi:10.3389/fnins.2020.625844)
Supplement: Supplementary file 1 [file Data_Sheet_1.docx]

The correlations between plasma fibrinogen with amyloid-beta and tau levels in patients with Alzheimer’s disease

**Dong-Yu Fan^1#^, Hao-Lun Sun^1,2#^, Pu-Yang Sun^1^, Jie-Ming Jian^1^, Wei-Wei Li^1^, Ying-Ying Shen^1^, Fan Zeng^1^, Yan-Jiang Wang^1*^, Xian-Le Bu^1*^**

^1^Department of Neurology and Centre for Clinical Neuroscience, Daping Hospital, Third Military Medical University, Chongqing, China.

^2^Shigatse Branch, Xinqiao Hospital, Third Military Medical University, Shigatse, China.

# These authors contributed equally to this work.

*** Correspondence:**Dr. Xian-Le Bu, email: [buxianle@sina.cn](mailto:buxianle@sina.cn); and Dr. Yan-Jiang Wang, email: [yanjiang_wang@tmmu.edu.cn](mailto:yanjiang_wang@tmmu.edu.cn)


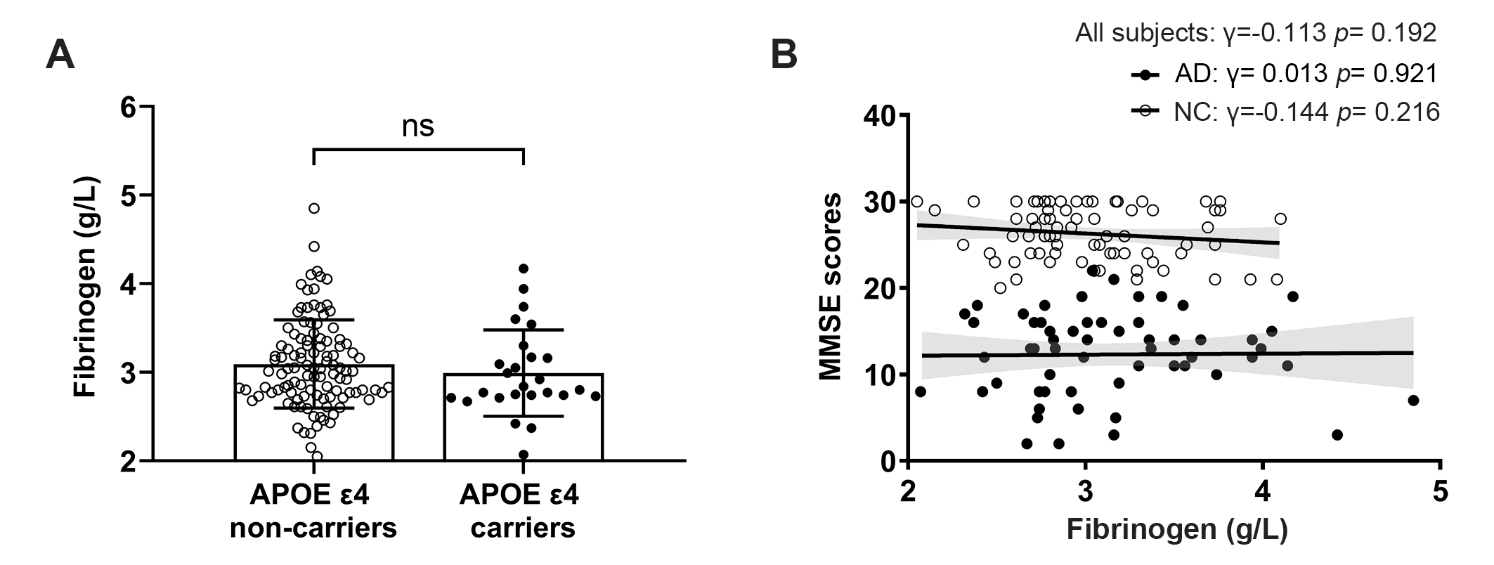


**Supplemental figure 1.** Comparison of the plasma fibrinogen levels in different APOE ε4 status (A). Correlations between fibrinogen levels with MMSE scores in AD patients diagnosed by positive PiB-PET and normal controls (NC) (B). ns denotes no statistical significance.


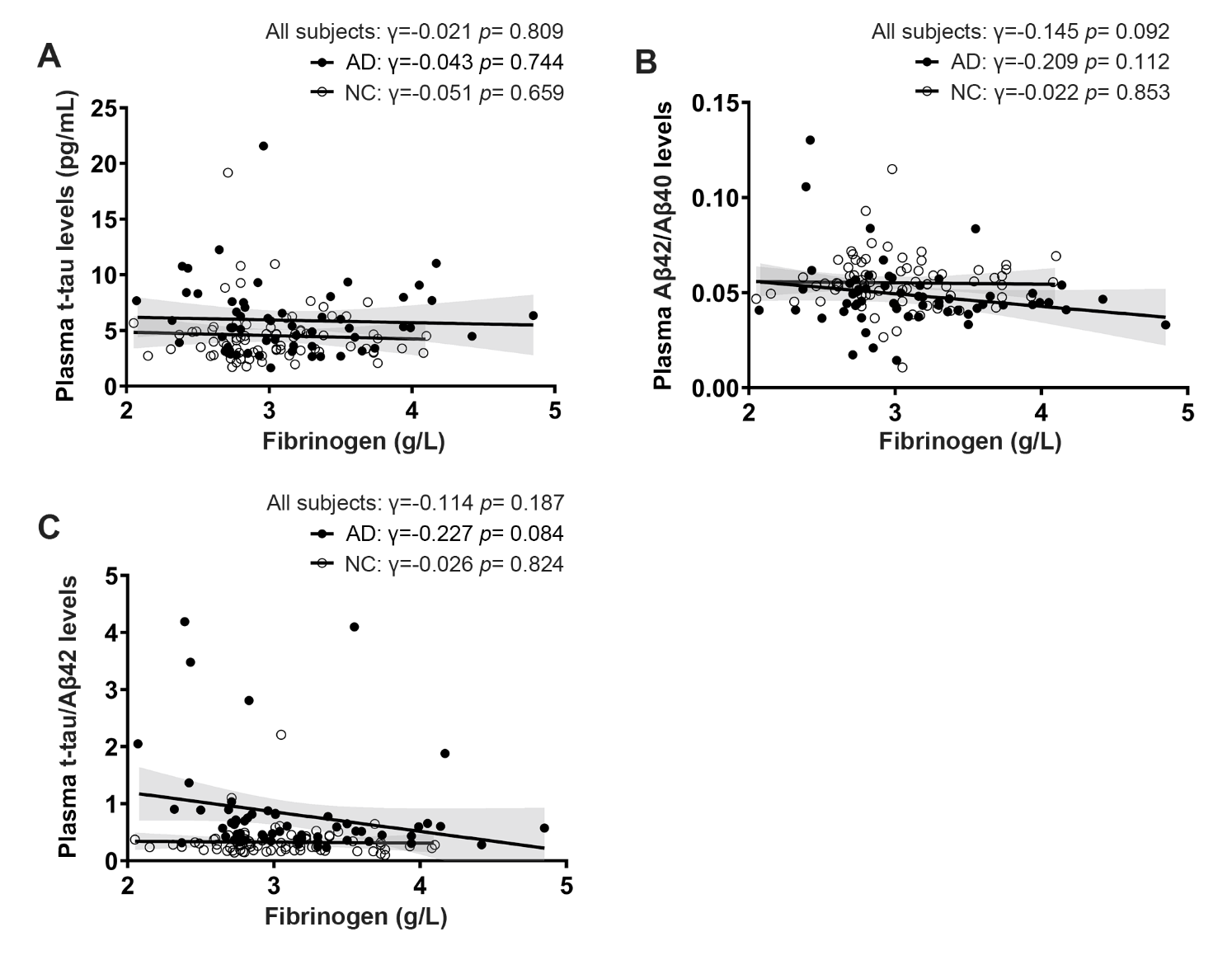


**Supplemental figure 2.** Correlations between fibrinogen levels with plasma t-tau levels (A), Aβ42/Aβ40 levels (B), and t-tau/Aβ42 levels (C) in AD patients and normal controls (NC).
